# Pathological variants in HPV-independent vulvar tumours

**DOI:** 10.1038/s41598-024-84688-3

**Published:** 2025-01-09

**Authors:** Sanja A. Farkas, Alvida Qvick, Gisela Helenius, Gabriella Lillsunde-Larsson

**Affiliations:** 1 Department of Laboratory Medicine, Clinical Pathology and Genetics, Faculty of Medicine and Health, Örebro University, Örebro, Sweden; 2 Clinical Research Center, Faculty of Medicine and Health, Örebro University, Örebro, Sweden; 3 ATMP Center, Skåne University Hospital, Lund, Sweden; 4 School of Health Sciences, Örebro University, Örebro, Sweden

**Keywords:** Vulvar squamous cell cancer, HPV, Comprehensive genetic profiling, Cancer genetics, Tumour biomarkers

## Abstract

Vulvar cancer is a rare gynaecological disease that can be caused by infection with human papillomavirus (HPV). The mutational frequencies and landscape for HPV-associated and HPV-independent vulvar tumor development are supposedly two distinctly different pathways and more detailed knowledge on target biological mechanisms for individualized future treatments is needed. The study included formalin-fixed paraffin-embedded (FFPE) samples from 32 cancer patients (16 HPV-negative and 16 HPV-associated), treated in Örebro, Sweden from 1988 to 2008. The Oncomine™ Comprehensive Assay v3 was used to detect variants across 161 different tumor relevant genes. Data analysis included quality assessment followed by variant analysis of DNA with the Oncomine Comprehensive v3 workflow and with a custom filter using the VarSome Clinical software. The RNA-analysis was performed with the Oncomine Comprehensive v3 workflow. Totally, 94% of DNA libraries and 81% of RNA libraries were of adequate quality for further downstream analysis. With the Oncomine™ filter chain there was an increased number of variants in the HPV-negative group (2.5 variants) compared to the HPV-associated group (1.5 variants). Using custom filter and the Varsome Clinical software; additional single nucleotide variants (SNV) were detected where the vast majority were classified as likely benign/benign. HPV-negative tumors had a larger fraction of variants of unknown significance (VUS), and likely pathogenic/pathogenic compared to the HPV-associated tumours. The top 10 frequently mutated genes in HPV-indepentent tumors were *TP53*,* POLE*,* PTCH1*,* BRCA2*,* CREBBP*,* NOTCH2*,* ARID1A*,* CDKN2A*,* MSH2*, and *NOTCH1.* Three fusion genes were detected; TBL1XR1(1)::PIK3CA(2) (*n* = 2) and NF1(5)::PSMD11(2) (*n* = 1). Copy number variations (CNV) were more common in HPV-associated tumors (*n* = 13/16, 81%) compared to HPV-negative tumors (*n* = 9/14, 64%). The most frequent CNV was found in the *cMYC* gene, followed by *CDK2* (*n* = 5) and *CDK4* (*n* = 4). The main outcome of this study show that vulvar cancer harbour genetic variations of different types and specifically, HPV-independent tumours are molecularly very heterogeneous and harboured more SNVs while HPV-associated tumors more frequently presented with gene amplifications. The PI3K/AKT/mTOR1 pathway was affected in both the groups as well as the cell cycle regulation pathway. Similarly, the DNA repair gene *POLE* was found mutated in both vulvar cancer groups.

**Supplementary Information:**

The online version contains supplementary material available at 10.1038/s41598-024-84688-3.

## Introduction

Vulvar cancer is a rare gynaecological disease caused either by Human papillomavirus (HPV) or by effects of chronic inflammation. Despite being a rare condition, the occurrence is increasing in many western countries^1–3^, possibly due to increased lifespan and increased HPV spread. Women carrying the disease are vulnerable and treatment will affect their sexual health in many ways.

Recently, the WHO classification of the disease was changed from morphology criteria to HPV-positive and HPV-negative^4^. The two groups have shown to have different risk assessment where women with HPV-independent tumours in some studies have been shown to have a worse prognosis^5–7^. However, as for cervical cancer, not all women with HPV-infection or chronic inflammation will develop cancer. Besides HPV, mutational and epigenetic events are necessary to alter cell functions, induce genomic imbalance, and drive a lesion towards carcinogenesis^8^.

The mutational frequencies and landscape for HPV-associated and HPV-independent tumour development are supposedly two distinctly different pathways and early studies have revealed p53 alterations as drivers for HPV-independent tumors^9^. Recently, the methodological improvement of next generation sequencing (NGS) has provided tools for looking closely into mutated genes, gene fusions and copy number alterations (CNVs).

For HPV-associated vulvar cancer, others and we have shown that HPV 16 is the most common genotype^10,11^. HPV 16, due to its oncogenic potential, is also the most frequently found genotype in cervical cancer, vaginal cancer, penile cancer and cancer of the head- and neck^12^. Several HPV 16- related factors have been investigated in relation to prognostic relevance, and for vulvar cancer we have previously shown that both a high viral copy number as well as high methylation of HPV 16 E2- binding sites were associated with worse cancer-specific survival rate^13^.

With more detailed knowledge on target biological mechanisms, vulvar cancer patients could potentially be given individual treatment and monitoring. Here we aim to evaluate two etiologically different groups of vulvar cancers to study the mutational pattern on both DNA as well as RNA- level with focus on the pathogenicity of the identified variants.

## Materials and methods

### Patient samples

This study included patients with vulvar squamous cell cancer (VSCC). The samples were collected between the years 1988–2008 and included in the study based on the HPV-status (HPV-positive or HPV-negative). The median age of the patients in the HPV-positive group was lower compared to the HPV-negative group, however it was not statistically significant (Table 1). The clinical findings were examined by a pathologist (Table 1). The HPV-genotypes found in the samples were HPV 16 (*n* = 13), HPV 33 (*n* = 1), HPV 56 (*n* = 1) and HPV 59 (*n* = 1)^11^. All samples were formalin-fixed paraffin-embedded (FFPE), and the tumour cell content ranged between 25 and 75%^11^.

**Table 1:** Patient characteristics with vulvar squamous cancer.

Characteristic	HPV-associated n = 16		HPV- negative n = 16		*p*-value*	Total n = 32	
Age					0.105^a^		
Median, (range)	65	(52–89)	77	(49–95)		71	(49–95)
Histology, n (%)					0.752^b^		
Keratinizing	7	(43.8)	11	(68.8)		18	(56.3)
Basal cell	6	(37.5)	1	(6.3)		7	(21.9)
Mixed	3	(18.8)	4	(25.0)		7	(21.9)
Stage, n (%)					0.155^b^		
I	3	(18.8)	1	(6.3)		4	(12.5)
II	7	(43.8)	7	(43.8)		14	(43.8)
III	4	(25.0)	6	(37.5)		10	(31.3)
IV	2	(12.5)	2	(12.5)		4	(12.5)
Tumor cell content (%)					0.0053^a^		
Median, (range)	25	(25–75)	50	(40–75)			

* HPV – associated vs. HPV – negative.

^a^ Mann-Whitney U test.

^b^ Chi-Square test.

**Table 2:** Location and type of variants detected in HPV-associated and HPV-negative vulvar tumours after filtering with the custom filter chain.

			Type of variants detected			
HPV-status	Total (N)	Variants in CDS	SNV	Substitution^1^	Deletion	Insertion
HPV-negative						
D1000	151	39	146	3	2	0
D1001	192	55	183	3	3	3
D1002	1835	1155	1807	24	3	1
D1003	165	46	157	2	4	2
D1006	172	90	172	0	0	0
D1007	177	87	171	1	4	1
D1008	160	44	153	2	2	3
D1009	163	38	155	3	4	1
D1010	158	37	149	1	6	2
D1011	164	43	158	1	1	4
D1012	170	43	163	2	2	3
D1013	143	33	134	1	2	6
D1014	174	45	167	2	2	3
D1015	497	238	484	7	5	1
Mean	309	142	300	4	3	2
Median	167,5	44,5	160,5	2	2,5	2
Total	4321	1993 (46%)	4199 (97,2%)	52 (1,2%)	40 (0,9%)	30 (0,7%)
HPV-associated						
D1016	123	36	119	1	3	0
D1017	138	40	129	3	4	2
D1018	131	36	123	2	5	1
D1019	265	113	252	5	5	3
D1020	131	37	124	2	4	1
D1021	165	44	159	1	3	2
D1022	162	48	156	1	2	3
D1023	182	48	171	2	6	3
D1024	137	33	128	2	6	2
D1025	163	46	156	1	6	0
D1026	148	45	142	3	2	1
D1027	182	53	175	0	6	1
D1028	160	43	158	1	1	0
D1029	140	36	133	1	3	3
D1030	161	37	158	0	2	1
D1031	145	40	138	3	3	1
Mean	158	46	151	2	4	2
Median	154	41,5	149	1,5	3,5	1
Total	2533	735 (29%)	2421 (95,6%)	28 (1,1%)	61(2,4%)	24 (0,9%)

CDS, coding DNA sequence.

N, number.

SNV, single nucleotide variant.

^1^More than one nucleotide variant has been changed.

**Table 3:** Number and percentage (%) of variants analysed with varsome clinical tool after filtering with the custom filter chain.

HPV status	Total (*N*)	Number of variants classified as				
		Benign	Likely benign	VUS	Likely pathogenic	Pathogenic
HPV-negative						
D1000	151	131	12	5	0	3
D1001	192	181	5	5	0	1
D1002	1835	170	932	578	107	48
D1003	165	151	7	4	1	2
D1006	172	38	73	38	13	10
D1007	177	93	42	33	5	4
D1008	160	144	8	3	1	4
D1009	163	129	23	8	1	2
D1010	158	152	2	2	1	1
D1011	164	153	3	3	0	5
D1012	170	165	3	1	0	1
D1013	143	136	5	1	1	0
D1014	174	164	5	3	0	2
D1015	497	151	201	118	20	7
Total	4321	1958 (45%)	1321 (31%)	802 (19%)	150 (3.5%)	90 (2.1%)
HPV-associated						
D1016	123	111	7	4	1	0
D1017	138	123	9	6	0	0
D1018	131	124	5	1	1	0
D1019	265	142	79	37	5	2
D1020	131	123	4	3	1	0
D1021	165	160	4	1	0	0
D1022	162	156	3	2	0	1
D1023	182	167	7	5	0	3
D1024	137	130	2	4	1	0
D1025	163	155	3	3	1	1
D1026	148	138	5	4	1	0
D1027	182	171	4	4	1	2
D1028	160	155	3	2	0	0
D1029	140	129	3	4	0	4
D1030	161	153	5	2	1	0
D1031	145	135	4	6	0	0
Total	2533	2272 (90%)	147 (5.8%)	88 (3.5%)	13 (0.5%)	13 (0.5%)

N, number.

VUS, variant of unknown significance.

**Table 4:** Top ten frequently mutated genes in our cohort of HPV-negative vulvar tumours compared to corresponding gene frequency in HPV-associated cases and to other studies.

Gene	Percentage of VSCC with the gene alteration in present cohort		Percentage of VSCC with the gene alteration in other studies		References
	HPV-negative	HPV-associated	HPV-negative	HPV-associated	
*TP53*	86%	0%	41–93%	0–9%	^14–22^
*POLE*	50%	6%			
*NOTCH1*	43%	19%	23–47%	NA	^15,18,19^
*CDKN2A*	36%	0%	9–55%	0–9%	^14,17–20,22^
*NOTCH2*	21%	0%			
*MSH2*	21%	0%			
*PTCH1*	14%	0%			
*BRCA2*	14%	0%	NA	7%	^35^
*CREBBP*	14%	12%			
*ARID1A*	7%	6%			

NA, not addressed.

### DNA and RNA extraction

All cases were sectioned in parallel, 1 × 10 μm each, for DNA and RNA extraction. The nucleic extraction was performed on the MagLEAD 12gc (Precision System Science, Mainz, Germany) using the FFPE DNA/RNA Purification kit (Precision System Science). DNA and RNA quantification was performed on the Qubit 2.0 Flurometer using the Qubit dsDNA HS Assay Kit and Qubit RNA HS assay kit, respectively (ThermoFisher Scientific, Waltham, MA).

### Library preparation and sequencing

The Oncomine™ Comprehensive Assay v3 (ThermoFisher Scientific) was used to detect variants across 161 different tumor relevant genes by analysing samples with a DNA-panel (SNV, indels and CNV detection) and RNA-panel (fusion detection), see Supplementary Table 1 for entire gene list. DNA and RNA libraries were prepared using the Ion Ampliseq™ DL8 Chef-Ready Library preparation kit. For DNA libraries, 75ng of template DNA was used in 18 amplification rounds with 8 min of annealing and extension. Prior to RNA library amplification cDNA was synthesized using 133–735 ng of RNA (SuperScript VILO cDNA Synthesis Kit, ThermoFisher Scientific). RNA libraries were synthesized with 31 amplification cycles holding 4 min of annealing and extension. Libraries were quantified with Ion Library Taqman™ Quantitation kit on 7500 Fast Real-Time PCR System according to the user guide.

The RNA and DNA libraries were diluted to 50pM and pooled in a 1:1 ratio prior to template preparation using the Ion Chef™ instrument according to the manufacturer’s instructions (Ion 540 ^TM^ - MAN0010851, revision D). The sequencing was performed using the Ion GeneStudio™ S5 Prime System.

### Data analysis

The data quality assessment of the sequenced libraries was performed based on the measurements of mapped reads, mean depth, uniformity and alignment over a target region obtained from Torrent Server™ or Ion Reporter™ Software (v. 5.10). DNA libraries were analysed using the Oncomine Comprehensive v3 workflow DNA (w4.1 – single sample) by two approaches, the first was using the Oncomine™ filter chain and excluded variants with a variant allele frequency < 5%, and a coverage < 300 reads. The second approach was to apply a custom filter chain to include more variants and asses the pathogenicity according to ACMG guidelines with the VarSome Clinical (Version11.6) software. The following parameters were set for the custom filter: Filtered Coverage < = 299; 0.05 < = Allele Ratio < = 1.0; Variant Type in INDEL, LOH, LONGDEL, MNV, SNV and Variant Effect in missense, nonframeshiftInsertion, nonframeshiftDeletion, nonframeshiftBlockSubstitution, nonsense, stoploss, frameshiftInsertion, frameshiftBlockSubstitution, frameshiftDeletion, nonsense. The derived sequences were aligned to DNA reference library hg19 (Human(hg19)).

The RNA-analysis was performed with the Oncomine Comprehensive v3 Fusions (w3.2 – single sample) workflow with modifications in fusion sensitivity that was set to low and the minimum of number of mapped reads was set to 100 000 reads. The RNA-fusion detection is based on known fusion break-points between driver genes and fusion genes in the human genome.

**Fig. 1:**
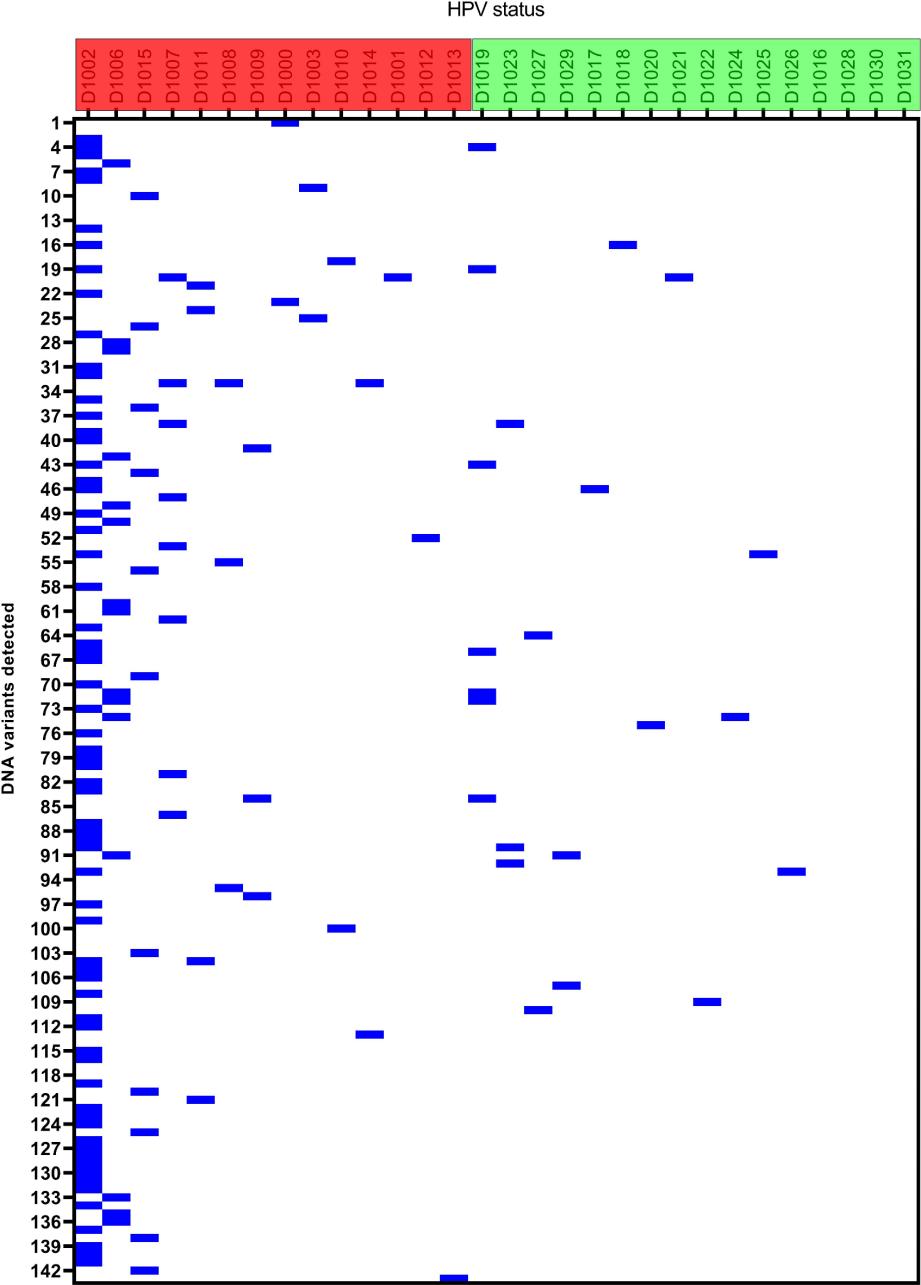
The figure shows all SNV, MNV and indels detected with the oncomine filter and 5% variant allele frequency threshold. Each row corresponds to a variant and a column to an individual sample. Some samples in the HPV-negative group (red) harboured more variants compared to the HPV-associated tumors (green).

**Fig. 2:**
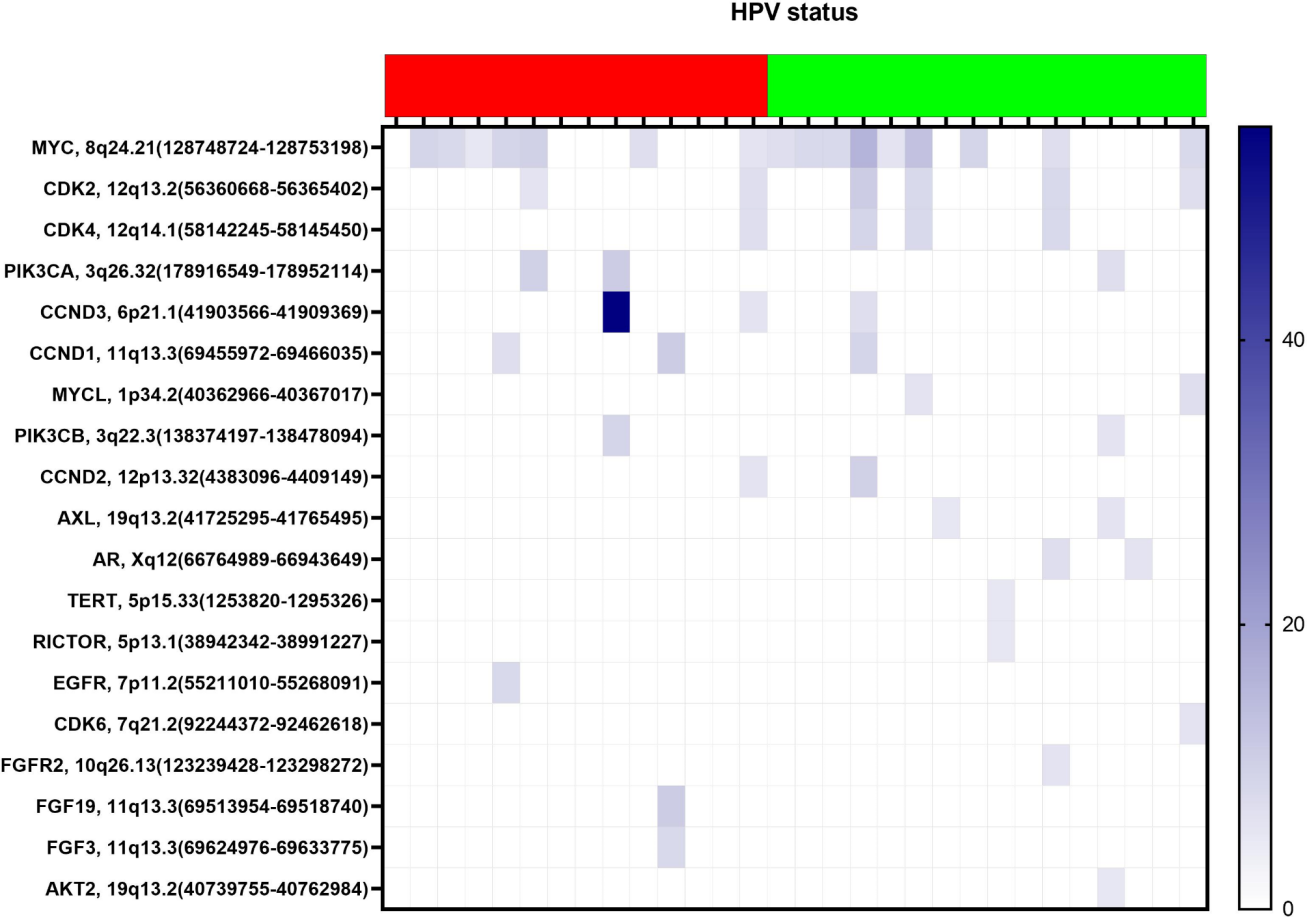
The figure shows all CNV:s detected in the HPV-negative (red) and HPV-associated group (green). The blue colour indicates the presence of a CNV and the depth of the colour the number of copies.

### Ethical approval

The study was approved by the regional ethical committee board in Uppsala, Sweden (Dnr 2008/294 and with approved amendment 2010-01-25). Specific informed consent from patients was not required, according to the ethical approval. Patients were verbally informed about the clinical research database. Patients were also informed about tissue biobanking in accordance with the Swedish Biobank Act 2002:297. The study was conducted in accordance with the Declaration of Helsinki.

### Statistics

Calculations of mean values and figures were done in excel spreadsheet, GraphPad Prism 9 (v. 9.3.1) or IBM SPSS Statistics version 29.0.0.0(241).

## Results

### Quality assessment

The sequenced samples (*n* = 32) were quality assessed prior variant annotation and interpretation. For DNA, samples were excluded if the sequenced library had a mean depth < 300 reads and uniformity values < 80% (*n* = 2). In total, 14 HPV-negative cases and 16 HPV-associated cases were analyzed with the OCA v3 DNA- panel and included for further variant annotation (Supplementary Fig. 1 and supplementary Fig. 2) - The mean sequencing depth was 2146 reads (range 321–4242) for HPV-negative group and 2531 reads (range 1725–4174) for the HPV-associated group and was considered adequate. The median mapped reads on target was 95% for all the samples, supplementary Fig. 3 shows the range.

For RNA, samples were excluded if the library had < 100 000 mapped reads(*n* = 6) In total, 10 HPV-negative cases and 16 HPV-associated cases were analyzed with the OCA v3 RNA-panel and included for further analysis. The mean sequencing depth was 1808487 reads (range 410219–5480227 reads) for samples in the HPV-negative group and 1535603 reads (range 426020–3688105 reads) for samples in the HPV-associated group.

### Detected genetic variants

Unfiltered data showed on average 3887 variants per sample, and the HPV-negative tumours had more variants compared to HPV-associated tumours (Supplementary Table 2). The Oncomine™ filter chain was used to narrow the list of variants identified in the analysis, based on public and proprietary annotation source from Ion Reporter. In short, it calls variants that have a gene or a variant class annotation such as loss-of function, gain of function or hot spot. Applying this filter chain, the median number of variants per sample were 2.5 in the HPV-negative group compared to 1.5 variants per sample in the HPV-associated group (Supplementary Table 2). Three cases in the HPV-negative group had ≥ 10 variants (Fig. 1). They belonged to the keratinizing histology group and tumor stage IA - III. Totally, 143 unique variants were detected with *Oncomine filter chain* variant annotation in the 30 vulvar cancer cases (Fig. 1). The Oncomine filter chain was found to be too stringent, and to evaluate more in depth the type of variants present in the data set we created a custom filter chain with the parameters allele ratio, coverage, and variant type. When applying the custom filter chain, the median number of variants increased to 168 in the HPV-negative group and 165 variants in the HPV-associated group (Supplementary Table 2).

Three samples carried fusion genes. The TBL1XR1(1)::PIK3CA(2) fusion was found in two cases: one HPV-associated and one HPV-negative case, and the NF1(5)::PSMD11(2) fusion was found in one HPV-positive case.

CNV were detected in both the HPV-negative and positive group of tumors. However, more HPV-associated tumors (*n* = 13/16, 81%) presented CNVs compared to HPV-negative tumors (*n* = 9/14, 64%), and eleven cases had more than one CNV (see Fig. 2). The most frequent CNV was found in the *cMYC* gene (*n* = 16), followed by *CDK2* (*n* = 5) and *CDK4* (*n* = 4).

### Variant classification using varsome clinical

Variants filtered with the custom filter chain were analysed with Varsome Clinical software (Supplementary Table 2). The most common nucleotide base changes were G > A and C > T nucleotide changes (Supplementary Table 3). The majority of the variants were located in the non-coding region in the genome (54% for HPV-associated and 70% for the HPV-negative cases) and SNV was the most common variant type (Table 2).

The variants were classified according to ACMG-guidelines using the Varsome Clinical software, and the vast majority of the variants were classified as likely benign/benign (Table 3). The HPV-associated tumors had a larger proportion of benign/likely benign variants compared to HPV-negative tumors (96% and 76%, respectively). The HPV-negative tumours had a larger proportion of variants of unknown significance (VUS), and likely pathogenic/pathogenic compared to the HPV-associated tumours (VUS: 19% and 3,5%; likely pathogenic/pathogenic: 3,5% and 1%, respectively).

Applying the custom filter chain, the most top 10 mutated genes in HPV-negative group were *TP53*,* POLE*,* PTCH1*,* BRCA2*,* CREBBP*,* NOTCH2*,* ARID1A*,* CDKN2A*,* MSH2*, and *NOTCH1*, and were classified as likely pathogen/pathogen (Table 4 and Supplementary Table 4). The top 10 mutated genes in the HPV-associated tumours were *PTEN*,* NOTCH1*,* PIK3CA*,* CREBBP*,* NF1*,* FANCA*,* POLE*,* ARID1A*,* MLH1*,* and ATRX.*

## Discussion

In the present study, we addressed the genetic changes in vulvar cancer analysing both DNA and RNA to explore the frequency of benign/likely benign, VUS, and likely pathogenic/pathogenic variant classes. In relation to other recent studies using similar approaches, our findings of mutated genes reflect what others have shown with some exceptions. Others have previously described mutations in *TP53*,* CDKN2A* and *NOTCH1* in vulvar cancers^14–22^ and accordingly *TP53* mutation is the most common variant finding in our HPV-negative tumors, also known as the HPV-independent tumors.

The mutational profile of HPV-associated tumors in the current study was considerably different from HPV-independent cases. Among the top ten mutated genes, none of the identified mutated genes were more frequent in HPV-associated tumors compared to HPV-independent tumors. Besides *TP53*, mutations in *POLE*,* NOTCH1*/*2* as well as *CDKN2A* were common in HPV-independent tumors (21–50%). *NOTCH1*, *CREBBP*, *ARID1A* and *POLE* were mutated in some of the HPV-associated tumors (6–19%).

In our cohort, HPV-positivity and *TP53* mutations were mutually exclusive. This finding was also true for studies by Corey et al.^14^, Prieske et al.^17^, and Han et al.^21^. However, in some concurrent studies, *TP53* mutations were evident in both HPV-independent and HPV-associated cases, but often found with lower frequencies^15,18,19,22^. Not all HPV-independent tumors in the present study harbored *TP53* mutations but those cases (*N* = 2) are supposedly affected by other genetic events. In the present study, one of the HPV-independent and *TP53* wild-type case had a pathological variant in the *NOTCH1* gene, and the other case harbored multiple other pathological variants. Studies have revealed worse outcomes in HPV-independent vulvar cancer compared to HPV-associated cancer^5–7^, but further molecular information may render knowledge on both subgroup of tumors and also provide therapeutic insights. Treatment in vulvar cancer is most often surgery but may be concurrent with radiation and chemotherapy, especially in advanced tumors with poor prognosis^23^. Advanced vulvar cancer with metastatic spread remains difficult, but a subgroup of patients may be candidates for mutation specific targeted therapy and clinical trials.

Molecular targets and pathways of therapeutic interest include both HPV-associated and HPV-independent tumors. Mutations within cell cycle regulation include p53 and pRB inactivation, as well as mutations within the family of cyclins. A loss of p53 function leads to uncontrolled cellular proliferation due to abrogated cell cycle arrest. For HPV-associated cases loss of p53 function is related to viral oncoprotein degradation of p53 while in HPV-independent cases, *TP53* mutations are frequent. Cyclins together with cyclin-dependent kinases (CDK) direct cell- cycle phase migration and are also important tumor suppressor players in cell cycle regulation^24^. Alterations in *CDKN2A* has been reported in several studies as potential oncogenic driver^14,17–20,22,25^. Others also report this finding to be unique for HPV-independent cases^14,17,19^, and often concurrent in cases that are *TP53* mutated^14,17^. Double mutated *TP53* and *CDKN2A* have been speculated to be extremely unfavorable in penile cancer^26^. In our study, 36% of HPV-independent tumours were found double-mutated, and the loss of these two tumor suppressors might be explored further in larger cohorts.

Mutational frequency has been found to be higher in vulvar HPV-independent cancers compared to HPV-associated tumors^15,18,19,26,27^, a phenomenon also recognized in head- and neck tumors^28^. *NOTCH* mutations were frequently seen in our cohort, as well as in other studies including vulvar cancer cohorts^15,18,19^, but also in penile cancer^26,27^. The NOTCH pathway induces transcription of target genes and starts with signals from NOTCH receptors at cell membrane levels. Important for cell to cell signaling, NOTCH signaling is dysregulated in many cancers affecting different mechanisms. For example, NOTCH has been suggested to act both as oncogene and tumor suppressor in head and neck cancer^29^. Also, mutant *NOTCH1* has been shown to activate PI3K-AKT-mTOR1 signaling, another pathway that is often more associated with HPV-associated cancer^30^. *POLE* mutations were the second largest finding among HPV-independent cases (50%). Also, among HPV-associated cases some tumors were mutated (6%). Priske et al.^17^ and Han et al.^21^ reported a subset of tumors also being mutated in *POLE*, however to a much lesser extent. Pole is a subunit of DNA polymerase epison and involved in DNA replication and repair. It has shown to be a prognostic marker in endometrial cancer^31–33^ but not common in other gynecological malignancies. This rather substantial amount of *POLE* mutated tumors in this cohort is a novel finding and important to follow up in larger cohorts and relate to prognostic variables.

The number of CNVs differed between HPV-associated and HPV-independent tumours. Amplifications where more common in HPV-associated tumours, with 81% of the tumours affected in one or more genes compared to 64% of the HPV-negative tumours. The most frequent found CNV was *cMYC*, a proto-oncogene coding for transcription factors that affect many functions important for tumor development. *cMYC* has found to be overexpressed in several cancer types, for example cervical cancer, potentially due to integration of viral genome^34^. *cMYC* amplification is reported sparsely in previous studies on vulvar cancer, Xing and colleagues^19^ also report this finding, especially among HPV-independent cases. However, amplifications in *PIK3CA* and *TERT* are more frequently reported^18,21,35^. A gain in *cMYC* has also been reported for penile cancer^36^ and in anal squamous cell cancer^37^ indicating the role in other squamous cell carcinomas. Besides *cMYC*,* CDK2* and *CDK4* was found to be amplified the current study. *CDK2* is a partner protein to MYC, aiding MYC-dependent regulation of genes that control senescence^38^.

Apart from SNVs and CNVs, gene fusion events are another important class of alterations in cancers. In the present study, we assessed the presence of gene fusion events with a gene panel including 51 genes. Three cases presented gene fusion events, two had a TBL1XR1(1)::PIK3CA(2) and one a NF1(5)::PSMD11(2) fusion. *PIK3CA* fusion events are less common compared to mutations, but could be an additional oncogenic mechanism and have previously been detected in breast and prostate cancer^39^. *NF1* gene is a known tumour suppressor gene and fusion events may lead to gene inactivation^40^. A previous study using RNA-sequencing has described three different fusion events in vulvar SCC: STIP1::CREB3L1, ZDHHC5::GPR137, and CELF1::DDIAS^41^. Another study addressing pathological changes in adenoid cystic cancer in the vulva, a very rare form of cancer, the MYB::NF1B fusion events were detected in 33% of cases^42^. Fusion events might be various and rare in vulvar cancer but still a mechanism that contributes to the oncogenicity of the tumour.

The main outcome of this study show that the vulvar SCC harbour genetic variations of different types. The HPV-independent group harboured more SNVs in comparison to HPV-associated tumors that more frequently presented with gene amplifications. We speculate that the presence of integrated HPV might contribute to an increase of gene amplification events. The PI3K/AKT/mTOR1 pathway was affected in both the groups either by the presence of SNVs or gene fusions. The cell cycle regulation pathway genes *TP53* och *CDKN2A* were mutated only in the HPV-negative group while the *MYC*,* CDK2* and *CDK4* were mutated in both groups. Similarly, the DNA repair gene *POLE* was found mutated in both SCC vulvar cancer groups. Precision medicine is changing the oncology field and treatment strategies from tumor-specific to molecular-specific therapies^43,44^. Patients with vulvar HPV-independent cancer harbour a variety of pathological mutations and this specific group of patients would most likely benefit from the molecular-specific approach if included in clinical trials studying various biomarkers such as gene mutations, gene fusions or other complex genetic biomarkers such as microsatellite instability. A limitation of our study is that our cohort included mainly HPV-16 positive cases. Vulvar SCC is a rare disease and the inclusion of cases with various HPV-types is challenging, future studies investigating the variant frequency in vulvar carcinomas with other HPV-types are needed. Samples from HPV-independent cases tended to have lower quality resulting in lower sequencing depth. This could have affected the sensitivity of variants with low VAF leading to an underrepresentation of the number of variants in this group. We acknowledge that an additional limitation is that the sequencing panel is limited to calling SNV/CNV in regions targeted by the assay, however we find it is valuable for the scope of this study. The used panel design includes genes most frequently mutated in various solid tumours and is suitable for the analysis of specimen with sparse material.

In conclusion, the present study added further knowledge in the characterization of the genomic changes in vulvar carcinoma that is a very rare form of cancer, and identified various pathological mutations in HPV-independent cancers. Our study together with others, suggests that HPV-independent tumours are molecularly very heterogeneous. There are various pathogenic mechanisms involved in each specific tumor, indicating that the patients would benefit from a molecular-specific therapy, also known as tumor-agnostic.

## Electronic supplementary material

Below is the link to the electronic supplementary material.

Supplementary Material 1.

## Data Availability

The datasets used and/or analysed during the current study are available from the corresponding author on reasonable request. The data are not publicly available because of patient confidentiality and ethical restrictions. Please contact the corresponding author regarding requests of original data.

## Abbreviations

CNV: Copy number variant
FFPE: Formalin fixed paraffine embedded
HPV: Human papillomavirus
SNV: Single nucleotide variant
VSCC: Vulvar squamous cell carcinoma
