# Supplementary material for: Pathological variants in HPV-independent vulvar tumours
Source: Sci Rep. 2025 Jan 9;15:1486. doi: 10.1038/s41598-024-84688-3 (PMC11718117; doi:10.1038/s41598-024-84688-3)
Supplement: Supplementary file 1 — Supplementary Material 1. [file 41598_2024_84688_MOESM1_ESM.docx]

**Pathological variants in HPV-independent vulvar tumours**

Sanja A. Farkas, Alvida Qvick ^1, 2^, Gisela Helenius ^2^, and Gabriella Lillsunde-Larsson ^1, 2^.

1. Department of Laboratory Medicine, Clinical Pathology and Genetics, Faculty of Medicine and Health, Örebro University, Örebro, Sweden.
2. School of Health Sciences, Örebro University, Örebro, Sweden.

**Supplementary Figures**


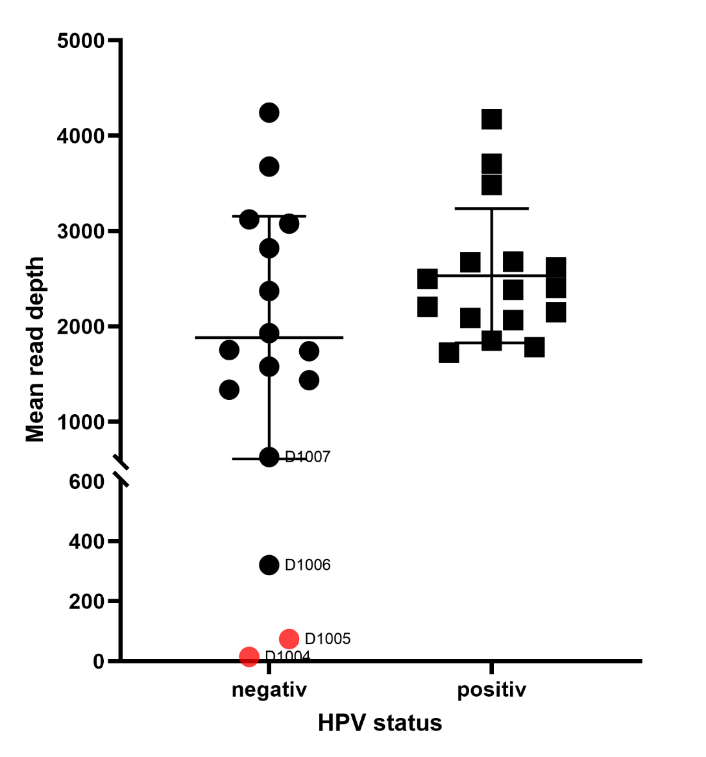


**Supplementary figure 1.** Mean read depth per sample in the HPV negative (
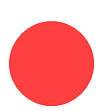
 ) and HPV positive (
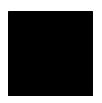
) group when analysed with the OCA v3 DNA-panel. The red colour indicates samples that have < 300 reads in mean depth and therefore have been excluded from further analysis.


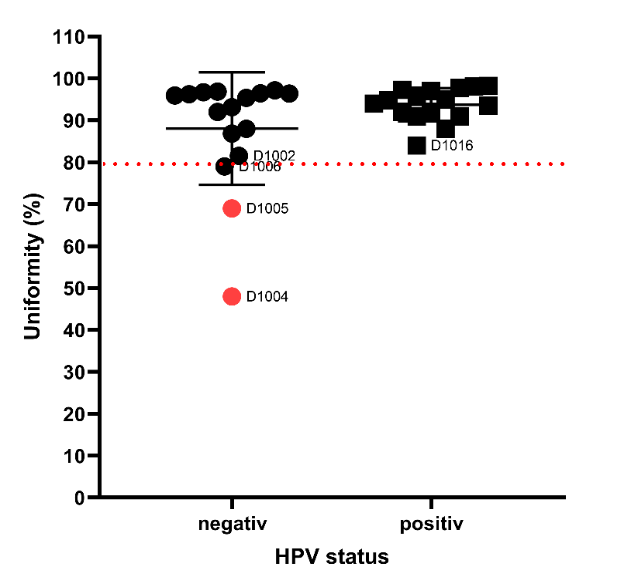


**Supplementary figure 2.** Sequencing uniformity per sample in the HPV negative (
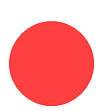
 ) and HPV positive (
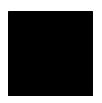
) group when analysed with the OCA v3 DNA-panel. The red colour indicates samples that have a uniformity < 80% and therefore have been excluded from further analysis.

**Supplementary figure 3.** Mapped reads on target shown for each sample in the HPV-negative and HPV-positive group when analysed with the OCA v3 DNA-panel.

**Supplementary Tables**

**Supplementary table 1.** Specific genes covered by the Oncomine comprehensive assay v3.

| **Hot-spot genes** | |  |  |  |  |  |
| --- | --- | --- | --- | --- | --- | --- |
| *AKT1* | *CSF1R* | *FLT3* | *JAK3* | *MTOR* | *PTPN11* | *TOP1* |
| *AKT2* | *CTNNB1* | *FOXL2* | *KDR* | *MYC* | *RAC1* | *U2AF1* |
| *AKT3* | *DDR2* | *GATA2* | *KIT* | *MYCN* | *RAF1* | *XPO1* |
| *ALK* | *EGFR* | *GNA11* | *KNSTRN* | *MYD88* | *RET* |  |
| *AR* | *ERBB2* | *GNAQ* | *KRAS* | *NFE2L2* | *RHEB* |  |
| *ARAF* | *ERBB3* | *GNAS* | *MAGOH* | *NRAS* | *RHOA* |  |
| *AXL* | *ERBB4* | *H3F3A* | *MAP2K1* | *NTRK1* | *ROS1* |  |
| *BRAF* | *ERCC2* | *HIST1H3B* | *MAP2K2* | *NTRK2* | *SF3B1* |  |
| *BTK* | *ESR1* | *HNF1A* | *MAP2K4* | *NTRK3* | *SMAD4* |  |
| *CBL* | *EZH2* | *HRAS* | *MAPK1* | *PDGFRA* | *SMO* |  |
| *CCND1* | *FGFR1* | *IDH1* | *MAX* | *PDGFRB* | *SPOP* |  |
| *CDK4* | *FGFR2* | *IDH2* | *MDM4* | *PIK3CB* | *SRC* |  |
| *CDK6* | *FGFR3* | *JAK1* | *MED12* | *PIK3CA* | *STAT3* |  |
| *CHEK2* | *FGFR4* | *JAK2* | *MET* | *PPP2R1A* | *TERT* |  |
|  |  |  |  |  |  |  |
|  |  |  |  |  |  |  |
| **Full-length genes** | |  |  |  |  |  |
| *ARID1A* | *FANCI* | *POLE* | *TP53* |  |  |  |
| *ATM* | *FBXW7* | *PTCH1* | *TSC1* |  |  |  |
| *ATR* | *MLH1* | *PTEN* | *TSC2* |  |  |  |
| *ATRX* | *MRE11* | *RAD50* |  |  |  |  |
| *BAP1* | *MSH6* | *RAD51* |  |  |  |  |
| *BRCA1* | *MSH2* | *RAD51B* |  |  |  |  |
| *BRCA2* | *NBN* | *RAD51C* |  |  |  |  |
| *CDK12* | *NF1* | *RAD51D* |  |  |  |  |
| *CDKN1B* | *NF2* | *RNF43* |  |  |  |  |
| *CDKN2A* | *NOTCH1* | *RB1* |  |  |  |  |
| *CDKN2B* | *NOTCH2* | *SETD2* |  |  |  |  |
| *CHEK1* | *NOTCH3* | *SLX4* |  |  |  |  |
| *CREBBP* | *PALB2* | *SMARCA4* |  |  |  |  |
| *FANCA* | *PIK3R1* | *SMARCB1* |  |  |  |  |
| *FANCD2* | *PMS2* | *STK11* |  |  |  |  |
|  |  |  |  |  |  |  |
| **Copy number genes** | | |  |  |  |  |
| *AKT1* | *ERBB2* | *MYC* |  |  |  |  |
| *AKT2* | *ESR1* | *MYCL* |  |  |  |  |
| *AKT3* | *FGF19* | *MYCN* |  |  |  |  |
| *ALK* | *FGF3* | *NTRK1* |  |  |  |  |
| *AXL* | *FGFR1* | *NTRK2* |  |  |  |  |
| *AR* | *FGFR2* | *NTRK3* |  |  |  |  |
| *BRAF* | *FGFR3* | *PDGFRA* |  |  |  |  |
| *CCND1* | *FGFR4* | *PDGFRB* |  |  |  |  |
| *CCND2* | *FLT3* | *PIK3CB* |  |  |  |  |
| *CCND3* | *IGF1R* | *PIK3CA* |  |  |  |  |
| *CCNE1* | *KIT* | *PPARG* |  |  |  |  |
| *CDK2* | *KRAS* | *RICTOR* |  |  |  |  |
| *CDK4* | *MDM2* | *TERT* |  |  |  |  |
| *CDK6* | *MDM4* |  |  |  |  |  |
| *EGFR* | *MET* |  |  |  |  |  |
|  |  |  |  |  |  |  |
|  |  |  |  |  |  |  |
| **Gene fusions** | |  |  |  |  |  |
| *AKT2* | *ETV5* | *NRG1* | *RELA* |  |  |  |
| *ALK* | *FGFR1* | *NTRK1* | *RET* |  |  |  |
| *AR* | *FGFR2* | *NTRK2* | *ROS1* |  |  |  |
| *AXL* | *FGFR3* | *NTRK3* | *RSPO2* |  |  |  |
| *BRCA1* | *FGR* | *NUTM1* | *RSPO3* |  |  |  |
| *BRCA2* | *FLT3* | *PDGFRA* | *TERT* |  |  |  |
| *BRAF* | *JAK2* | *PDGFRB* |  |  |  |  |
| *CDKN2A* | *KRAS* | *PIK3CA* |  |  |  |  |
| *EGFR* | *MDM4* | *PRKACA* |  |  |  |  |
| *ERBB2* | *MET* | *PRKACB* |  |  |  |  |
| *ERBB4* | *MYB* | *PTEN* |  |  |  |  |
| *ERG* | *MYBL1* | *PPARG* |  |  |  |  |
| *ESR1* | *NF1* | *RAD51B* |  |  |  |  |
| *ETV1* | *NOTCH1* | *RAF1* |  |  |  |  |
| *ETV4* | *NOTCH4* | *RB1* |  |  |  |  |

**Supplementary table 2.** Number of DNA-variants detected in vulvar squamous cell cancer with the Oncomine Comprehensive assay and various filtering strategies.

| **Number of variants identified** | | | |
| --- | --- | --- | --- |
|  | **Without filter chain** | **Oncomine filter chain^1^** | **Custom filter chain^2^** |
| ***HPV-negative*** | | | |
| D1000 | 3311 | 2 | 151 |
| D1001 | 3399 | 1 | 192 |
| D1002 | 10443 | 66 | 1835 |
| D1003 | 3487 | 2 | 165 |
| D1006 | 7455 | 15 | 172 |
| D1007 | 5335 | 8 | 177 |
| D1008 | 3388 | 3 | 160 |
| D1009 | 3585 | 3 | 163 |
| D1010 | 3214 | 2 | 158 |
| D1011 | 3263 | 4 | 164 |
| D1012 | 3237 | 1 | 170 |
| D1013 | 3218 | 1 | 143 |
| D1014 | 3232 | 2 | 174 |
| D1015 | 5792 | 11 | 497 |
| *Mean* | *4454* | *9* | *309* |
| *Median* | *3394* | *2,5* | *168* |
| ***HPV-associated*** | | | |
| D1016 | 3473 | 0 | 123 |
| D1017 | 3529 | 1 | 138 |
| D1018 | 3333 | 1 | 131 |
| D1019 | 4913 | 7 | 265 |
| D1020 | 3229 | 1 | 131 |
| D1021 | 3262 | 1 | 165 |
| D1022 | 3235 | 1 | 162 |
| D1023 | 3290 | 3 | 182 |
| D1024 | 3198 | 1 | 137 |
| D1025 | 3296 | 1 | 163 |
| D1026 | 3320 | 1 | 148 |
| D1027 | 3248 | 2 | 182 |
| D1028 | 3245 | 0 | 160 |
| D1029 | 3207 | 2 | 140 |
| D1030 | 3233 | 0 | 161 |
| D1031 | 3241 | 0 | 145 |
| *Mean* | *3391* | *1,4* | *158* |
| *Median* | *3378* | *1,5* | *165* |
| Total (all tumours) | 166611 | 143 | 6854 |

^1^ Oncomine filter chain and AF >5%, reads > 300

^2^ Custom filter: Filtered Coverage >= 299; 0.05 <= Allele Ratio <= 1.0; Variant Type in FLT3ITD, INDEL, LOH, LONGDEL, MNV, SNV

**Supplementary table 3.** The number and percent (%) base changes that were detected in VSCC. Nucleotide base change C>T and G>A were most common in both the groups..

|  | A⇒C | A⇒G | A⇒T | C⇒A | C⇒G | **C⇒T** | **G⇒A** | G⇒C | G⇒T | T⇒A | T⇒C | T⇒G | Indels | Total |
| --- | --- | --- | --- | --- | --- | --- | --- | --- | --- | --- | --- | --- | --- | --- |
| HPV-associated | 68 (1,6%) | 255 (5,9%) | 54 (1,2%) | 47 (1,1%) | 124 (2,9%) | 1509 (34,9%) | 1516 (35,1%) | 100 (2,3%) | 51 (1,2%) | 39 (0,9%) | 409 (9,5%) | 27 (0,6%) | 122 (2,8%) | 4321 |
| HPV-negative | 90 (3,6%) | 296 (11,7%) | 76 (3,0%) | 53 (2,1%) | 150 (5,9%) | 487 (19,2%) | 512 (20,2 %) | 132 (5,2%) | 54 (2,1%) | 56 (2,2%) | 475 (18,8%) | 40 (1,6%) | 112 (4,4%) | 2533 |

**Supplementary table 4.** Specific variants detected in the top 10 mutated genes in HPV-negative patients.

| **Chomosome, koordinate and variant** | | | **HPV-negative** | **HPV-positive** |
| --- | --- | --- | --- | --- |
| *TP53,* NM_000546.6 | |  | 15 |  |
| chr17 | 7574034 | c.994-1G>C | 1 |  |
| chr17 | 7577094 | c.844C>T(p.Arg282Trp) | 1 |  |
| chr17 | 7577099 | c.839G>C(p.Arg280Thr) | 1 |  |
| chr17 | 7577105 | c.833C>T(p.Pro278Leu) | 1 |  |
| chr17 | 7577120 | c.818G>A(p.Arg273His) | 1 |  |
| chr17 | 7577520 | c.761T>C(p.Ile254Thr) | 1 |  |
| chr17 | 7577538 | c.743G>A(p.Arg248Gln) | 2 |  |
| chr17 | 7577539 | c.742C>T(p.Arg248Trp) | 1 |  |
| chr17 | 7578263 | c.586C>T(p.Arg196Ter) | 1 |  |
| chr17 | 7578290 | c.560-1G>A | 1 |  |
| chr17 | 7578392 | c.538G>A(p.Glu180Lys) | 1 |  |
| chr17 | 7578437 | c.493C>T(p.Gln165Ter) | 1 |  |
| chr17 | 7578500 | c.430C>T(p.Gln144Ter) | 1 |  |
| chr17 | 7579313 | c.374C>T(p.Thr125Met) | 1 |  |
| *POLE*, NM_006231.4 | |  | 10 | 1 |
| chr12 | 133202807 | c.6427C>T(p.Gln2143Ter) | 1 |  |
| chr12 | 133210837 | c.5939G>A(p.Trp1980Ter) | 1 |  |
| chr12 | 133218984 | c.4953-1G>A | 1 |  |
| chr12 | 133235880 | c.3275+1G>A | 4 | 1 |
| chr12 | 133240735 | c.2562-1G>A | 1 |  |
| chr12 | 133245216 | c.2026+5G>A | 1 |  |
| chr12 | 133249341 | c.1558C>T(p.Gln520Ter) | 1 |  |
| *CDKN2A* | NM_000077.5 | | 6 |  |
| chr9 | 21974497 | c.330G>A(p.Trp110Ter) | 1 |  |
| chr9 | 21971108 | c.250G>A(p.Asp84Asn) | 1 |  |
| chr9 | 21971029 | c.329G>A(p.Trp110Ter) | 1 |  |
| chr9 | 21971120 | c.238C>T(p.Arg80Ter) | 3 |  |
| *NOTCH1*, NM_017617.5 | | | 6 | 3 |
| chr9 | 139405655 | c.2536C>T(p.Gln846Ter) | 1 |  |
| chr9 | 139399459 | c.4683_4684del(p.Ala1562GlyfsTer47) | 1 |  |
| chr9 | 139400282 | c.4053_4066del(p.Cys1352LeufsTer48) | 1 |  |
| chr9 | 139396350 | c.5488dup(p.Val1830GlyfsTer4) | 1 |  |
| chr9 | 139397633 | c.5167+1G>A | 1 |  |
| chr9 | 139412744 | c.1100G>A(p.Gly367Asp) | 1 |  |
| chr9 | 139396314 | c.5524C>T(p.Gln1842Ter) |  | 1 |
| chr9 | 139409132 | c.2037del(p.Ile679MetfsTer93) |  | 1 |
| chr9 | 139401195 | c.3874del(p.His1292ThrfsTer153) |  | 1 |
| *NOTCH2*, NM_024408.4 | | | 7 |  |
| chr1 | 120461029 | c.5929G>A(p.Gly1977Arg) | 1 |  |
| chr1 | 120465295 | c.4966C>T(p.Gln1656Ter) | 1 |  |
| chr1 | 120478212 | c.3538C>T(p.Gln1180Ter) | 1 |  |
| chr1 | 120467927 | c.4511+1G>A | 1 |  |
| chr1 | 120462975 | c.5356C>T(p.Arg1786Ter) | 1 |  |
| chr1 | 120465043 | c.5029C>T(p.Gln1677Ter) | 1 |  |
| chr1 | 120462851 | c.5479+1G>A | 1 |  |
| *PTCH1*, NM_000264.5 | |  | 9 |  |
| chr9 | 98239936 | c.1396C>T(p.Gln466Ter) | 1 |  |
| chr9 | 98244253 | c.724C>T(p.Gln242Ter) | 1 |  |
| chr9 | 98248001 | c.550C>T(p.Gln184Ter) | 1 |  |
| chr9 | 98248148 | c.403C>T(p.Arg135Ter) | 1 |  |
| chr9 | 98242849 | c.768G>A(p.Trp256Ter) | 1 |  |
| chr9 | 98244486 | c.585-1G>A | 1 |  |
| chr9 | 98221991 | c.2778G>A(p.Trp926Ter) | 1 |  |
| chr9 | 98247966 | c.584+1G>A | 1 |  |
| chr9 | 98221925 | c.2844G>A(p.Trp948Ter) | 1 |  |
| *BRCA2*, NM_000059.4 | | | 8 |  |
| chr13 | 32907124 | c.1509del(p.Lys503AsnfsTer6) | 1 |  |
| chr13 | 32936732 | c.7878G>A(p.Trp2626Ter) | 1 |  |
| chr13 | 32953937 | c.9004G>A(p.Glu3002Lys) | 1 |  |
| chr13 | 32953902 | c.8969G>A(p.Trp2990Ter) | 1 |  |
| chr13 | 32893463 | c.316+1G>A | 1 |  |
| chr13 | 32971106 | c.9573G>A(p.Trp3191Ter) | 1 |  |
| chr13 | 32954174 | c.9148C>T(p.Gln3050Ter) | 1 |  |
| chr13 | 32930582 | c.7453C>T(p.Gln2485Ter) | 1 |  |
| *CREBP*, NM_004380.3 | | | 9 | 2 |
| chr16 | 3786750 | c.4461C>G(p.His1487Gln) | 1 |  |
| chr16 | 3786748 | c.4463C>T(p.Pro1488Leu) | 1 |  |
| chr16 | 3786752 | c.4459C>T(p.His1487Tyr) | 1 |  |
| chr16 | 3828696 | c.1941+5G>A | 1 |  |
| chr16 | 3828712 | c.1930G>A(p.Ala644Thr) | 1 |  |
| chr16 | 3790511 | c.4022G>A(p.Arg1341Gln) | 1 |  |
| chr16 | 3820792 | c.2659C>T(p.Gln887Ter) | 1 |  |
| chr16 | 3781211 | c.5154G>A(p.Trp1718Ter) | 1 |  |
| chr16 | 3786776 | c.4435G>A(p.Gly1479Arg) | 1 |  |
| chr16 | 3781324 | c.5039_5041del(p.Ser1680del) |  | 1 |
| chr16 | 3929833 | c.85G>A(p.Asp29Asn) |  | 1 |
| *ARID1A*, NM_006015.6 | | | 6 | 1 |
| chr1 | 27097609 | c.3199-1G>A | 1 |  |
| chr1 | 27102131 | c.5057G>A(p.Trp1686Ter) | 1 |  |
| chr1 | 27087954 | c.2241_2242delCCinsTT(p.Gln748Ter) | 1 |  |
| chr1 | 27087961 | c.2248C>T(p.Arg750Ter) | 1 |  |
| chr1 | 27058033 | c.1741C>T(p.Gln581Ter) | 1 |  |
| chr1 | 27106307 | c.5918G>A(p.Trp1973Ter) | 1 |  |
| chr1 | 27099947 | c.3826C>T(p.Arg1276Ter) |  | 1 |
| *MSH2*, NM_000251.3 | |  | 6 |  |
| chr2 | 47635605 | c.277C>T(p.Leu93Phe) | 1 |  |
| chr2 | 47703564 | c.2064G>A(p.Met688Ile) | 1 |  |
| chr2 | 47703578 | c.2078G>A(p.Cys693Tyr) | 1 |  |
| chr2 | 47657041 | c.1237C>T(p.Gln413Ter) | 1 |  |
| chr2 | 47702265 | c.1861C>T(p.Arg621Ter) | 1 |  |
| chr2 | 47705577 | c.2377C>T(p.Gln793Ter) | 1 |  |
